# Supplementary material for: Can we infer excitation-inhibition balance from the spectrum of population activity?
Source: Commun Biol. 2025 Dec 13;9:51. doi: 10.1038/s42003-025-09315-x (PMC12795805; doi:10.1038/s42003-025-09315-x)
Supplement: Supplementary file 2 — Supplementary Information [file 42003_2025_9315_MOESM2_ESM.pdf]

Can we infer excitation-inhibition balance from the spectrum of population activity?

Kingshuk Chakravarty[1], Sangheeta Roy[1], Aniruddha Sinha[1], Arvind Kumar[2,3]

[1]*Tata Consultancy Services, Kolkata, India*
[2]*Division of Computational Science and Technology,*
School of Electrical Engineering and Computer Science, KTH Royal Institute of Technology Stockholm, Sweden
[3]*Science for Life Laboratory, Stockholm, Sweden*

Supplementary figures

Figure S1: Effect of input correlations on the aperiodic exponent and the variance of total synaptic conductance (as reflected in the LFP).

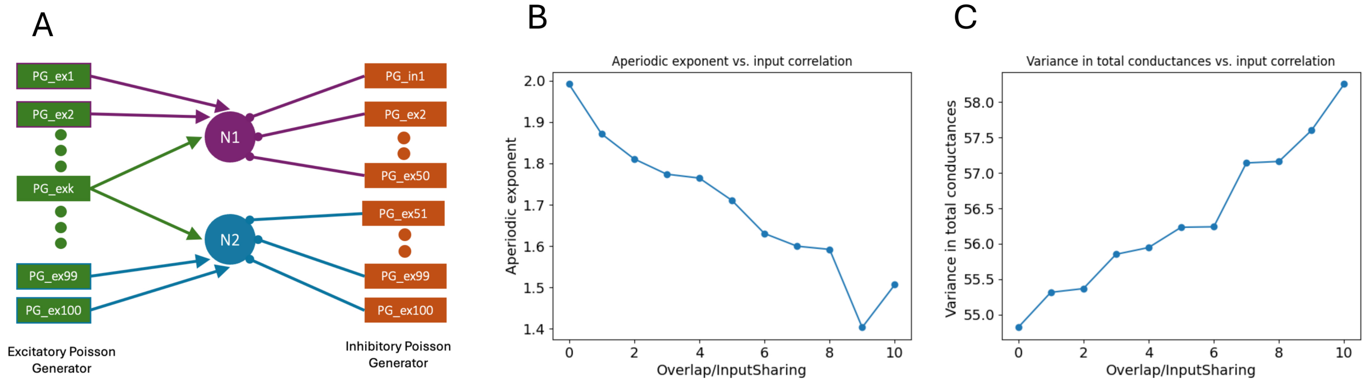

A. Schematic of the correlated inputs to the two neurons. Each neuron received Poisson type excitatory and inhibitory spike trains. Inhibitory inputs to each neuron came from independent sources. To control input correlations we varied the fraction of shared input. Correlation among excitatory inputs was controlled by varying the number of shared Poisson-type spike trains. Input correlation increases monotonically with the degree of shared (presynaptic) inputs between the neurons. B. Aperiodic component as a function of input sharing. C. Variance of simulated LFPs as a function of input sharing.

Figure S2: Comparison of OI_{avg} and λ_{avg} values derived by averaging of total conductances /LFPs across 10 neurons and 1000 neurons in the STN population.

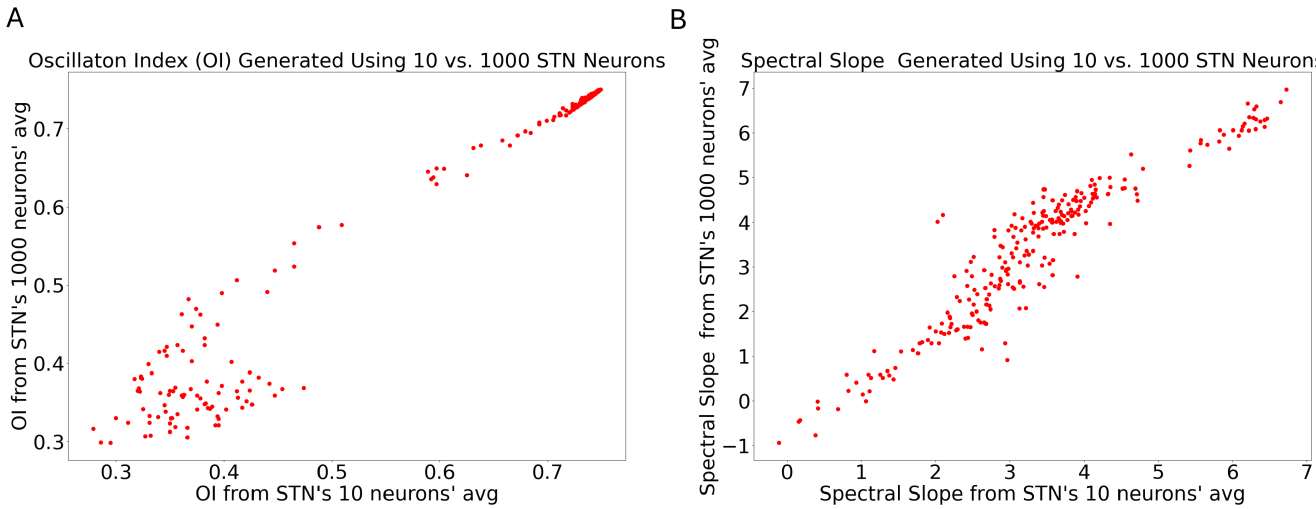

(A). Each dot represents OI_{avg} measured for 10 neurons (x-axis) or 1000 neurons (y-axis). This was done for a subset of 250 network configuration parameters selected randomly from a total of 625 network configurations. As is evident from this data, OI_{avg} estimates from 10 neurons is highly correlated with that estimates from 1000 neurons. (B.) Same as in panel A but for λ_{avg}. These data justify our choice of using 10 neurons for LFP generation in the manuscript. Note that same result holds for the neocortical networks.
